# Long non-coding RNA in glioma: signaling pathways

**DOI:** 10.18632/oncotarget.15175

**Published:** 2017-02-07

**Authors:** Jia Shi, Bo Dong, Jiachao Cao, Yumin Mao, Wei Guan, Ya Peng, Suinuan Wang

**Affiliations:** ^1^ Department of Neurosurgery, The Third Affiliated Hospital of Soochow University, Changzhou, China

**Keywords:** long non-coding RNAs, glioma, microRNA

## Abstract

Glioma is regarded as the most prevalent malignant carcinoma of the central nervous system. Thus, the development of new therapeutic strategies targeting glioma is of significant clinical importance. Long non-coding RNAs (lncRNAs) are functional RNA molecules without a protein-coding function and are reportedly involved in the initiation and progression of glioma. Dysregulation of lncRNAs in glioma is due to activation of several signaling pathways, such as the BRD4-HOTAIR-β-catenin/PDCD4, p53-Hif-H19/IGF2 and CRNDE/mTOR pathways. Furthermore, microRNAs (miRNAs) such as miR-675 also interact with lncRNAs in glioma. Thus, exploring the mechanisms by which lncRNA control processes will be instrumental for devising new effective therapies against glioma.

## INTRODUCTION

Glioma is the most common and aggressive malignant tumor of the central nervous system and has a high rate of recurrence and mortality [1]. According to the histological subtype and degree of malignancy, gliomas can be classified as astrocytomas, oligodendrogliomas, ependymomas and mixed tumors, with grades of I to IV [2]. Although there have been advances in multimodal treatments such as surgery, radiotherapy and chemotherapy [3], overall survival of most patients with glioma remains dismal, especially in cases of glioblastoma, for which the median survival time is only approximately 14 months [4].

**Figure 1:**
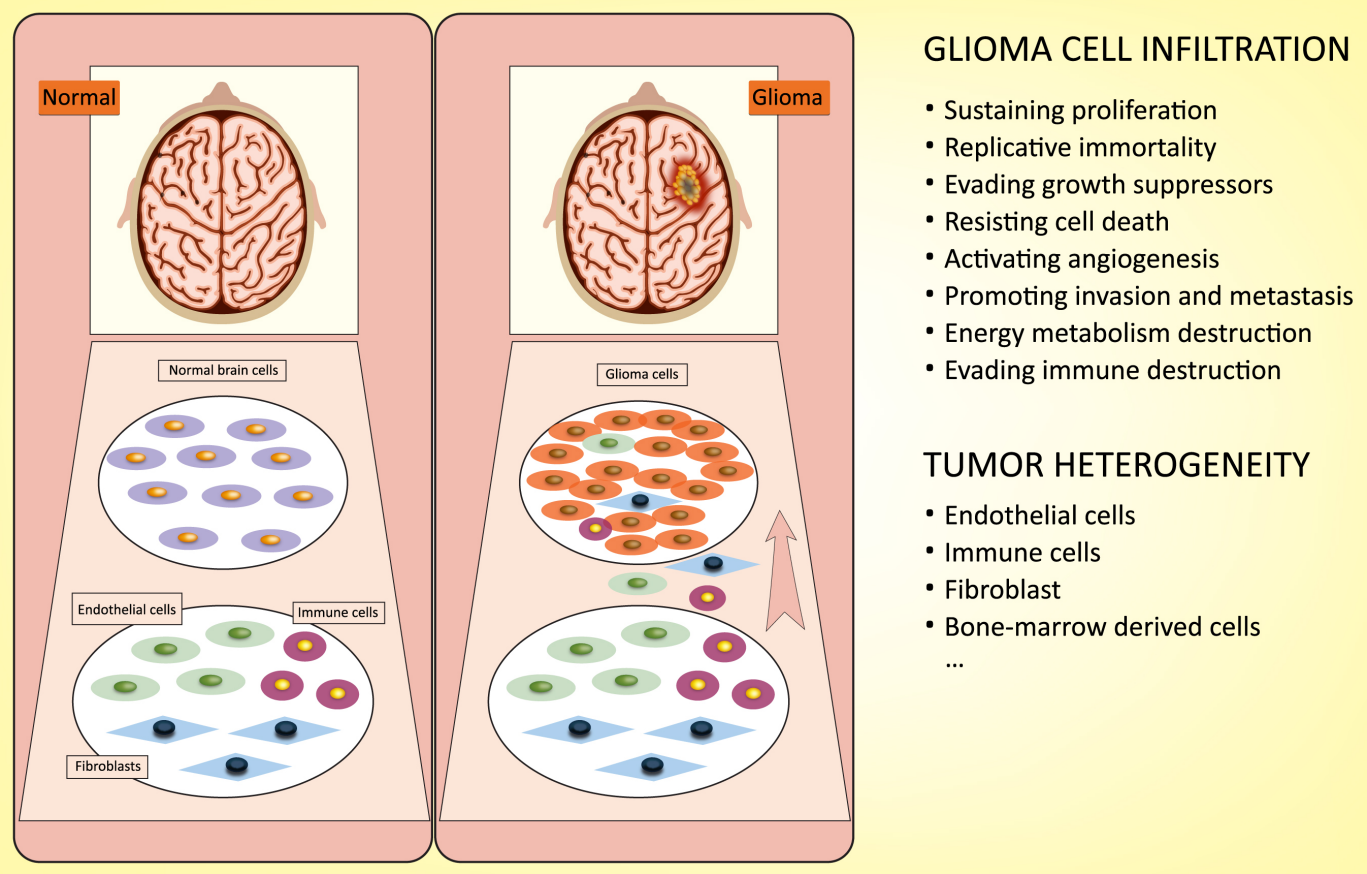
Characterization of glioma cells: infiltration and heterogeneity Infiltration: glioma cells exhibit a special growth pattern, involving sustaining proliferative activity, enabling replicative immortality, opposing growth suppressors, resisting cell death, activating angiogenesis, promoting invasion and metastasis, reprogramming energy metabolism and evading immune destruction. Heterogeneity: glioma cells recruit undifferentiated and differentiated cells to form a “tumor microenvironment” for tumorigenesis.

Tumor cell infiltration into normal brain tissue, which makes complete surgical excision challenging, is the main cause of the poor prognosis [5]. Compared to normal tissue, glioma cells exhibit an unbridled neoplastic growth pattern, which involves sustaining proliferative activity, enabling replicative immortality, opposing growth suppressors, resisting cell death, activating angiogenesis, promoting invasion and metastasis, reprogramming energy metabolism and evading immune destruction [6]. In addition, the heterogeneity of glioma cells makes them highly resistant to chemotherapy and radiotherapy [7, 8]. Glioma cells not only exhibit the characteristics listed above but also exhibit obvious heterogeneity by recruiting undifferentiated and differentiated cells to form a “tumor microenvironment” for tumorigenesis [9] (Figure 1). Therefore, there is a strong need for a better understanding of the occurrence and evolution of glioma at the genetic and molecular levels, and such knowledge will be beneficial for identifying new therapeutic strategies.

**Figure 2:**
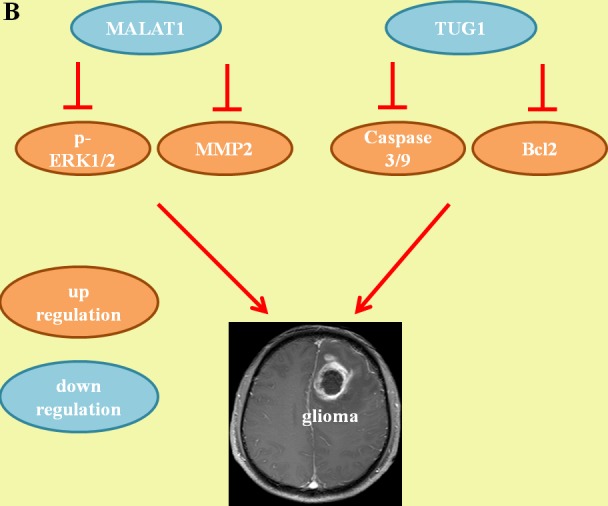

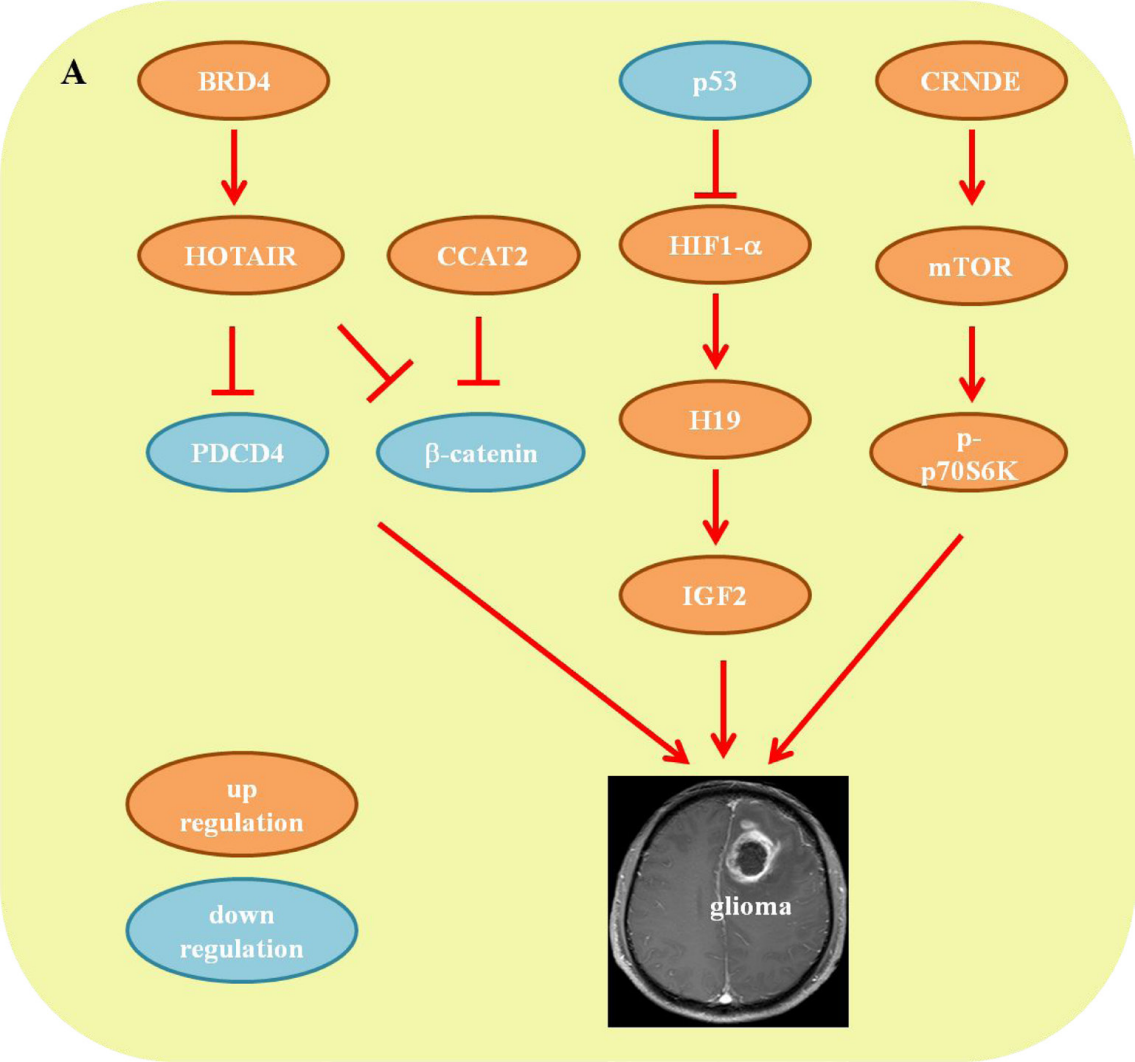
Mechanisms of lncRNA in glioma **A**. The pro-oncogenic lncRNA network. **B**. The tumor-suppressive lncRNA network.

### lncRNA

Long non-coding RNAs (lncRNAs) are non-protein-coding transcripts ranging from nearly 200 nucleotides (nt) to over 100 kilobases (kb) in length [10–12]. According to their different genomic locations and context, lncRNAs can be classified into five categories: sense, transcribed from the sense strand of protein-coding genes; antisense, transcribed from the opposite strand of mRNAs; intronic, transcribed from introns of protein-coding genes; intergenic, transcribed from intergenic regions; and bidirectional, transcribed from the vicinity of transcription start sites in both sense and antisense directions [13]. As transcriptional regulators, lncRNAs can alter gene transcription via transcriptional interference and chromatin remodeling [14, 15]. Moreover, lncRNAs can also alter gene expression post-transcriptionally by base pairing with translation factors or ribosomes to control translation or by binding to splicing factors to modulate splicing [16, 17]. Unlike microRNAs, which have been widely studied, little is known about the function of lncRNAs. However, evidence over the past 5 years has shown that lncRNAs have a significant function in tissue homeostasis and biological processes, including cancer progression [18].

Recently, several studies have indicated that aberrant expression of lncRNAs may affect glioma initiation and progression [19]; therefore, lncRNAs may act as biomarkers for glioma diagnosis, prognosis and target therapy [20]. In this review, we summarize the latest evidence for the roles of lncRNAs in regulating the biological behavior and the underlying mechanisms of glioma. Moreover, we provide an overview of the relationship between microRNA (miRNA) and lncRNA in glioma, which may lead to the identification of new therapeutic targets for this cancer.

### Differential lncRNA expression profiles in glioma

Dysregulation of lncRNA in glioma and the degree of malignancy have been investigated in several genome-wide profiling studies. Increased or decreased lncRNA expression results in a tumor suppressor or promoter role. Table 1 summarizes the aberrant expression of lncRNAs in glioma reported to date.

**Table 1:** Dysregulation of lncRNAs in glioma initiation and progression

lncRNA	Changes	Target	Ref.
HOTAIR1	up	β-catenin/PDCD4	[21, 22, 33–36]
CCAT2	up	β-catenin	[40]
CRNDE	up	mTOR	[21, 22, 52]
ASLNC22381	up	IGF1	[24]
ASLNC20819	up	IGF1	[24]
H19	up	IGF2	[27, 28, 44–49]
ENST00000545440	up		[26]
NR_002809	up		[26]
MALAT1	down	ERK-MAPK/MMP2/9	[55]
TUG1	down	Caspase3/9	[59, 60]
PART1	down		[25]
MIAT	down		[25]
GAS5	down		[25]
MEG3	down		[23]
C21orf131	down		[21]
BC002811	down		[26]
XLOC_010967	down		[26]
PAR5	down		[27, 28]
RFPL1S	down		[27, 28]

HOTAIR: HOX transcript antisense RNA; CCAT2: cancer-associated transcript 2; CRNDE: colorectal neoplasia differentially expressed; MALAT1: metastasis-associated lung adenocarcinoma transcript 1; TUG1: taurine up-regulated gene 1; PART1: prostate androgen-regulated transcript 1; MIAT: myocardial infarction associated transcript ; GAS5: growth arrest specific 5; MEG3: maternally expressed gene 3; PAR5: Prader Willi/Angelman region RNA 5; RFPL1S: RFPL1 antisense 1; β-catenin: catenin beta-1; PDCD4: programmed cell death protein 4; mTOR: mammalian Target of RapamycinIGF1: insulin growth factor 1; IGF2: insulin growth factor 2; ERK: extracellular signal–regulated kinases; MAPK: mitogen-activated protein kinase; MMP2/9: matrix metalloproteinase-2/9; Caspase3/9: cysteine-aspartic proteases.

### Dysregulation of lncRNA in glioma initiation

Recent microarray studies have revealed significant changes in the expression patterns of many lncRNAs in tissues of different subtypes of glioma and normal brain tissue. Using the lncRNA classification pipeline, Zhang et al. identified 1970 lncRNAs across different types and grades of human gliomas [21]. Similarly, Grzmil et al. conducted profiling analysis of 30 different glioma samples (12 primary glioblastoma multiforme (GBM), 3 secondary GBM, 8 astrocytomas and 7 oligodendrogliomas) and 5 GBM cell lines. The results showed that 147 of 2448 lncRNAs were differentially expressed in tumor tissues compared to normal tissues and that 213 lncRNAs were differentially expressed in tumor cell lines [22]. Intestinally, both studies confirmed the up-regulation of HOX transcript antisense RNA (HOTAIR) and colorectal neoplasia differentially expressed (CRNDE) in tumor samples. Wang et al. reported that maternally expressed gene 3 (MEG3) expression was markedly decreased in astrocytoma tissues compared to adjacent normal tissues. Moreover, MEG3 was positively associated with p53, and this association was necessary for activation of p53 [23].

GBM is the most common and aggressive type of primary brain tumor in humans. In a study by Han et al., 1308 lncRNAs were identified (654 up-regulated and 654 down-regulated) between GBM tissues and normal brain tissues (fold change ≥4.0 or ≤0.25, P<0.01). Further gene network analysis suggested that ASLNC22381 and ASLNC20819 are up-regulated by targeting insulin growth factor 1 (IGF1) in GBM recurrence and progression [24]. Zhang et al. also identified reduced expression of prostate androgen-regulated transcript 1 (PART1), myocardial infarction-associated transcript (MIAT) and growth arrest specific 5 (GAS5) in GBM tumors [25]. These findings suggest that dysregulation of lncRNA expression may be an early event during tumorigenesis, and these lncRNAs may have potentially important functions in glioma initiation.

### Dysregulation of lncRNA in the malignant progression of glioma

By comparing different lncRNA profiles between low-grade and high-grade astrocytoma, Zhang et al [21] identified 45 lncRNAs. Among these lncRNAs, 12 were found to be closely related to malignant progression. For example, expression of HOTAIR1 and CRNDE was enhanced with ascending tumor grade, though that of MEG3 and C21orf131 (LINC00320) was reduced [21]. In another study, Zhi et al. identified 7 differentially expressed lncRNAs between astrocytoma samples (World Health Organization WHO II-IV) and normal adjacent tissues using a commercial microarray platform. Unsupervised clustering analysis further demonstrated that up-regulation of ENST00000545440 and NR_002809 was positively associated with advanced clinical stages of astrocytoma. Moreover, Kaplan-Meier survival analysis showed that down-regulation of BC002811 and XLOC_010967 or up-regulation of NR_002809 was significantly associated with poor survival [26].

Other studies provide comparable evidence. For instance, Vital et al. investigated 46 lncRNAs differently expressed between 5 low-grade astrocytomas and 30 high-grade astrocytomas [27], and Zhou et al. described 48 lncRNAs differently expressed in 13 astrocytic gliomas (6 low grade and 7 high grade) [28]. Among these dysregulated lncRNAs, H19 expression increased with the tumor malignancy grade, whereas expression of Prader Willi/Angelman region RNA 5 (PAR5) and RFPL1 antisense 1 (RFPL1S) decreased with increasing tumor grade [27, 28]. These differential levels of lncRNA expression between malignancy grades indicate their involvement in glioma progression.

## MECHANISMS OF LNCRNA IN GLIOMA (TABLE 1)

### The pro-oncogenic lncRNA network

#### BRD4-HOTAIR-β-catenin/PDCD4

Bromodomain and extraterminal (BET) domain proteins are epigenetic modulators that have recently emerged as promising therapeutic targets in GBM [29–31]. Bromodomain containing 4 (BRD4), a BET protein, has been well-studied in glioma and has been demonstrated to bind to the HOTAIR promoter [32]. The lncRNA HOTAIR is identified as an oncogene in GBM; by interacting with polycomb repressive complex 2 (PRC2), HOTAIR is positively associated with glioma staging, poor prognosis and the molecular subtype of glioma [33]. Treatment with a BET inhibitor (I-BET151) decreases expression of HOTAIR and suppresses tumor cell proliferation by inducing cell cycle arrest. Conversely, overexpression of HOTAIR abrogates the anti-proliferative activity of I-BET151, a major target for GBM therapy [32]. Moreover, glioma stem cells (GSCs), a subpopulation of glioma cells, have important functions in sensitivity to chemoradiotherapy and the possibility of tumor recurrence [34]. Kan Fang et al. and Xuan Zhou et al. reported reducing HOTAIR expression inhibited GSC proliferation, induced cell cycle arrest, decreased cell invasive capacity and reduced tumorigenic capacity both *in vivo* and *in vitro*[35, 36]. The underlying mechanism was mediated via activation of programmed cell death protein 4 (PDCD4) expression at the transcriptional level [36] and repression of the β-catenin pathway [35]. The Wnt/β-catenin pathway was also shown to be associated with malignant progression and the poor prognosis of glioma patients [37–39]. Another lncRNA, cancer-associated transcript 2 (CCAT2), was found to be up-regulated in glioma tissues and positively correlated with tumor stage. Indeed, knockdown of CCAT2 inhibited glioma cell proliferation and migration by repressing the Wnt/β-catenin pathway [40]. Therefore, the findings with regard to BRD4-HOTAIR-β-catenin/PDCD4-mediated tumor development provide new insight into the complexity of glioma.

#### P53-HIF1-H19/IGF2

H19 is a 2.3-kb non-coding RNA that is widely expressed during embryonic development, which then decreases after birth [41]. Mounting evidence has demonstrated that expression of H19 is enhanced in both primary and metastatic tumors, including during morphogenesis and the epithelial-mesenchymal transition (EMT), and in inflammatory and multidrug-resistant tumors [42, 43]. The role of H19 in glioma has also recently been highlighted. Jiang X et al. reported that overexpression of H19 promotes the invasion, stemness, angiogenesis and tumorigenicity of glioblastoma cells, indicating the function of H19 in the tumorigenicity and stemness of glioma [44]. IGF2 is transcribed from four different promoters (P1-P4) and produces 4 different transcripts that share common regulatory sequences with H19, which is located within 200 kb downstream of the parental IGF2 gene at 11p.15.5 [45]. Several studies have shown that both IGF2-P4 transcripts and H19 are highly expressed in human glioblastoma cell lines and high-grade gliomas, affecting cell growth and motility [45, 46]. Moreover, hypoxia is a common feature of solid tumors, particularly malignant gliomas, and causes tumor cells to either die or exhibit a pleiotropic adaptive response by activating hypoxia-inducible transcription factor 1 (HIF1) [47]. Park et al. demonstrated that H19 expression was enhanced in p53 knockout mice upon hypoxia [48]; however, in the presence of functional p53, H19 was efficiently repressed via inhibition of HIF1-α transcriptional activity [49], demonstrating an important role of the p53-HIF1-H19 pathway in hypoxia. Additionally, IFG2 is also a target gene transactivated by HIF1 during the hypoxic response [47]. These studies highlight the p53-HIF-H19/IGF2 axis as a potential therapeutic target for glioma.

#### CRNDE/mTOR

lncRNA CRNDE, on chromosome 16 of the human genome, is the most up-regulated transcript in colorectal cancer [50]. Interestingly, a study by Zhang et al. showed that among 129 differentially expressed lncRNAs, CRNDE is also the most up-regulated lncRNA in glioma [21]. Moreover, correlation analysis demonstrated that the level of CRNDE is positively associated with tumor grade [22]. Mammalian Target of Rapamycin (mTOR) is a well-known regulator of cell growth and proliferation, and dysregulation of mTOR by either oncogene activation or tumor suppressor loss can induce tumor growth in various malignant cell lines [51]. Wang et al. reported that overexpression of CRNDE promotes cell growth and migration both *in vivo* and *in vitro* by increasing the phosphorylation level of p70S6K, a direct downstream target of mTOR. In contrast, CRNDE knockdown suppressed these processes by decreasing p70S6K phosphorylation, suggesting that CRNDE can influence mTOR signaling in glioma [52].

### The tumor-suppressive lncRNA network

#### MALAT1/MMP2/ERK/MAPK

The lncRNA metastasis-associated lung adenocarcinoma transcript 1 (MALAT1) is initially up-regulated in lung cancer, which is reported to be a favorable prognostic factor for patients with stage I non-small cell lung cancer [53, 54]. Recently, Han et al. reported reduced expression of MALAT1 in glioma tissues relative to that in normal brain tissues, with MALAT1 knockdown promoting cell proliferation and invasion both in human glioma cell lines and in glioma xenograft models. Conversely, overexpression of MALAT1 counteracted the significant repression of cell proliferation and invasion *in vitro* and *in vivo*, indicating a tumor-suppressive role for MALAT1 in glioma [55]. It is well known that tumor invasion and spread are the major causes of death in patients with glioma. The extracellular signal-regulated kinase/mitogen activated protein kinase (ERK/MAPK) pathway is one of the most important signal transduction pathways responsible for cell proliferation and invasion [56]. Matrix metalloproteinase 2 and 9 (MMP2 and -9) are also known to participate in cell invasion during enhanced tumor progression [57]. Mechanistic studies have demonstrated that overexpression of MALAT1 significantly decreases expression of phosphorylated ERK1/2 and MMP2/9 in U87 and U251 cells. Moreover, inhibition of ERK signaling (U0126) imitates MALAT1 overexpression-induced levels of phosphorylated ERK1/2 and MMP2, indicating that the tumor-suppressive function of MALAT1 is mediated by attenuating ERK/MAPK-mediated cell growth and MMP2/9-mediated invasiveness [55].

#### TUG1/caspase-3/caspase-9/Bcl2

Taurine up-regulated gene 1 (TUG1) is a 7.1-kb lncRNA that was initially detected in a genomic screen of retinal cells under taurine treatment [58]. The role of TUG1 in glioma pathogenesis was recently identified by several studies [59, 60]. Li et al. showed that expression of TUG1 was significantly inhibited in glioma, with negative correlations with tumor size, World Health Organization (WHO) grade and overall survival. Overexpression of TUG1 induces apoptosis in glioma cells by activating apoptotic genes caspase-3 and -9-mediated intrinsic pathways and inhibiting anti-apoptotic gene Bcl2-mediated anti-apoptotic pathways, suggesting a tumor suppressor role for TUG1 in human glioma [59].

## miRNA-lncRNA INTERACTION IN GLIOMA

miRNAs are 20-25 nt non-coding RNA molecules that act as repressors of gene expression by binding to partially complementary sequences in mRNA 3’ untranslated regions (UTRs) [61]. Distinct expression patterns and the functional significance of miRNAs have been reported for many cancers, including glioma [62]. miRNAs appear to be important regulators of GSC maintenance [63–65], invasiveness [64, 65], pathogenesis [66, 67] and epigenetic and signaling pathways [68, 69]. Recently, emerging evidence has linked miRNAs with lncRNAs in controlling glioma initiation and progression and has suggested that the miRNA-lncRNA interaction may provide novel insight into therapeutic targets for glioma (Table 2).

**Table 2:** MiRNA-lncRNA interactions in glioma

lncRNA	Changes	miRNA	Changes	Ref.
HOTAIR	up	miR-148b	down	[72]
		miR-326	down	[73]
H19	up	miR-675	up	[76, 77]
		miR-29a	up	[78]
CRNDE	up	miR-186	down	[81]
XIST	up	miR-152	down	[83, 84]
CASC2	down	miR-21	up	[89]

HOTAIR: HOX transcript antisense RNA; CRNDE: colorectal neoplasia differentially expressed; XIST: X-inactive specific transcript; CASC2: cancer susceptibility candidate 2.

Expression of miR-148b-3p, a member of the miR-148/152 family, is decreased in several tumor cell lines [70, 71]. Guan Wang et al. reported that miR-148b-3p suppresses glioma cell proliferation, cell cycle progression and invasion by inhibiting HOTAIR expression. Furthermore, it was demonstrated that miR-148b-3p binds to HOTAIR in a sequence-specific manner [72]. Additionally, HOTAIR can affect the malignant biological behavior of glioma by targeting miRNA. miR-326, which is down-regulated in both glioma samples and in two glioma cell lines (U87 and U251), showed a negative correlation with histopathological grades of glioma [73, 74]. Jing Ke et al. determined that HOTAIR promotes the development of glioma by inhibiting miR-326 and further promoting increased expression of fibroblast growth factor 1 (FGF1) [73], which has a significant oncogenic function in tumorigenesis through activation of the MEK1/2 and PT3K/AKT pathways [75].

MicroRNA-675 (miR-675-5p), a miRNA in the first exon of H19, is up-regulated in several cancer types, including glioma [76]. Yan Shi et al. and Chao Li et al. reported overexpression of H19 to be positively correlated with its derivate miR-675 in promoting glioma cell invasion and proliferation, though reduction of H19 inhibited miR-675 expression, thus abrogating carcinogenesis. Bioinformatics and luciferase reporter assays have further confirmed the underlying mechanisms; miR-675 inhibits cadherin 13 (Ca^2+^-dependent cell adhesion) or cyclin-dependent kinase 6 (CDK6) by directly targeting the binding site within the 3'UTR [76, 77]. Interestingly, as we demonstrated previously, hypoxia is considered a major driving force for glioma growth and angiogenesis. Alessia LD et al. showed that overexpression of miR-675-5p is sufficient to induce hypoxia under normoxia by increasing nuclear HIF-1α and its mRNA; moreover, depletion of miR-675-5p drastically abolishes hypoxia by decreasing levels of HIF-1α [77]. The H19-miR-675-HIF-1α loop network may provide a novel strategy for the hypoxia response in glioma. In addition to miR-675, miR-29a is also a potential target for H19 in glioma angiogenesis. Indeed, knockdown of H19 inhibits glioma and induces endothelial cell proliferation, migration and tube formation by decreasing expression of miR-29a. Additionally, miR-29a targets the 3'UTR region of vasohibin 2 (VASH2), which was identified as an angiogenic factor in tumor tissue [78].

miR-186 has been reported to be a tumor suppressor, and its expression is decreased in many tumors, such as esophageal cancer, lung adenocarcinoma and colorectal cancer [79, 80]. Recently, Zhang et al. showed that overexpression of CRNDE could promote GSC proliferation and invasion and inhibit apoptosis by suppressing miR-186. Mechanistic studies further revealed that miR-186 decreases expression of X-linked inhibitor of apoptosis (XIAP) and serine/threonine-protein kinase 7 (PAK7) by binding to their 3'UTR regions [81].

By inhibiting Kruppel-like factor 4 (Klf4), miR-152 is reported to be a tumor suppressor in human GSCs [82]. In a study by Yao et al., miR-152 was identified as a direct target of lncRNA X-inactive specific transcript (XIST), a product of the XIST gene, which regulates X inactivation in mammals [83, 84]. Knockdown of XIST exerted a tumor-suppressive function by decreasing GSC proliferation, migration and invasion as well as by promoting apoptosis. *In vivo* studies also showed that knockdown of XIST repressed tumor growth and induced high survival rates in nude mice. Knockdown of XIST also increased expression of miR-152, whereas overexpression of miR-152 reduced XIST expression and reversed the effects of XIST knockdown [84]. These results suggest that XIST and miR-152 may form a reciprocal repression feedback loop in glioma.

miR-21 is a well-known oncogene that is elevated in various malignant tumors, including glioma tissues and cell lines [85]. A growing body of literature has shown that knockdown of miR-21 inhibits tumorigenesis by regulating multiple factors associated with cell proliferation, migration, invasion and apoptosis [86, 87]. The lncRNA cancer susceptibility candidate 2 (CASC2), located on chromosome 10q26, acts as a tumor-suppressor in endometrial cancer [88]. Recently, Wang et al. found CASC2 to be down-regulated in glioma tissues and cell lines, though overexpression of CASC2 repressed glioma cell proliferation, malignancy and invasion and promoted apoptosis by directly inhibiting miR-21. Moreover, mechanistic analysis confirmed that miR-21 binds to CASC2 in a sequence-specific manner. In addition, introduction of miR-21 significantly repressed CASC2-mediated inhibition of glioma cell proliferation, migration, invasion and apoptosis, suggesting reciprocal repression between CASC2 and miR-21 [89].

## CONCLUSIONS

Recent studies of lncRNA and glioma have generated much interest in the field of cancer. Reports have demonstrated that lncRNAs exert significant biological functions in gliomas, including the initiation of malignancy, progression and other phenotypes. Here, we discussed dysregulation of lncRNA expression in glioma and the underlying mechanisms, which may potentially lead to the discovery of novel diagnostic and prognostic biomarkers.

However, some aspects need to be further explored. First, given that tumor cells exhibit obvious heterogeneity by recruiting various cells to form the tumor microenvironment [90], little is known about the relationship between lncRNAs and tumor heterogeneity. Second, although lncRNAs have been reported to participate in the progression of glioma, the stage at which lncRNAs function remains unknown. To address these issues, additional studies in large cohorts are needed to elucidate the specific and underlying regulatory pathways of lncRNAs in gliomas. Further knowledge of these lncRNAs may provide novel therapeutic strategies for glioma.
